# An Engineered Spike Immunogen Drives Broad Protection of a Bivalent Recombinant Protein COVID-19 Vaccine in Animal Models

**DOI:** 10.3390/v18090976

**Published:** 2026-09-04

**Authors:** Zikang Wang, Jiahua Gao, Ruojing Bai, Lunzhi Yuan, Huilin Guo, Shaojuan Wang, Youfeng Wang, Jiayi Wu, Qingfang Bu, Huiyu Guo, Yunda Hong, Jian Ma, Kai Wang, Wenjie Guo, Yanyan Liu, Zhitao Weng, Tianying Zhang, Ningshao Xia, Quan Yuan, Yali Zhang, Yangtao Wu

**Affiliations:** 1 State Key Laboratory of Vaccines for Infectious Diseases, Xiang An Biomedicine Laboratory, School of Public Health, Xiamen University, Xiamen 361102, China; 2 National Institute of Diagnostics and Vaccine Development in Infectious Diseases, Xiamen University, Xiamen 361102, China

**Keywords:** engineered Spike protein, bivalent vaccine, COVID-19, broad protection

## Abstract

The continued evolution of SARS-CoV-2 has reduced the protective effectiveness of first-generation vaccines and underscores the need for broadly reactive next-generation vaccine candidates. Here, we evaluated STFKB, an alum-adjuvanted bivalent recombinant protein vaccine composed of the monomeric Spike (STFK_prototype_) and the engineered Spike (STFK_1628X_). We assessed the immunogenicity, tolerability, and protective efficacy of STFKB in mice, rats, guinea pigs, rhesus macaques, and Syrian hamsters. STFKB induced robust STFK- and STFK_1628X_-specific antibody responses and broadly reactive neutralizing antibodies against multiple SARS-CoV-2 variants in the tested animal models. In hamster challenge studies, STFKB vaccination protected animals from Omicron BA.1 and BA.5 challenge, as shown by reduced body-weight loss, lower viral RNA loads in respiratory tissues, and improved gross lung pathology. Across the tested preclinical models, STFKB was well tolerated, with no vaccine-related overt toxicity observed under the study conditions. These findings support the rationale and translational potential of the bivalent vaccine strategy proposed in this study.

## 1. Introduction

Since the COVID-19 outbreak, SARS-CoV-2 has caused a global crisis, with over 779 million cases and 7 million deaths reported by March 2026 [1]. Viral entry is mediated by the spike (S) protein, specifically via its receptor-binding domain (RBD), which engages the ACE2 receptor [2,3]. This mechanism identifies the S protein as the primary target for vaccine and therapeutic development. Consequently, multiple vaccine platforms—including inactivated, live-attenuated, recombinant protein, VLP, mRNA, and viral vector vaccines—were rapidly developed [4,5,6,7,8,9,10,11,12,13,14,15]. Despite their diverse components and regimens, these vaccines have all made important contributions to disease prevention. The large-scale deployment of COVID-19 vaccines has been pivotal in mitigating viral spread and reducing the global disease burden.

Nevertheless, the continuous evolution of SARS-CoV-2 has introduced critical mutations (e.g., K417N, L452R) that have been reported to exert distinct effects on viral transmissibility, infectivity, and pathogenicity [16], posing substantial challenges to the effectiveness of existing vaccines [17]. Studies indicate that neutralizing antibody titers from primary regimens of inactivated, viral vector, and mRNA vaccines decline significantly against variants like Beta and Omicron—with reductions ranging from 2.5-fold to 42-fold compared to the prototype strain [18,19,20,21]. Even with booster immunization, persistent immune escape by variants like BA.4/5 remains a critical issue [22,23]. These findings underscore the urgent need to develop broadly protective vaccines that offer proactive cross-protection against a wider spectrum of future variants [24,25].

Despite the challenges of viral mutation [26], several promising strategies have emerged. Rational antigen design is central to this effort, utilizing chimeric antigens [27,28] and mosaic nanoparticles [29], and targeting conserved epitopes [30] and multivalent vaccines [31,32]. Notable examples include the mutI-tri-RBD vaccine, mosaic nanoparticles displaying heterologous RBDs, and authorized bivalent mRNA vaccines, all of which demonstrate enhanced breadth against multiple variants. Additionally, the development of novel adjuvants, such as selenium-based nanoparticles, has shown potential for broadly protective vaccines [33]. Furthermore, optimizing administration routes via mucosal (inhaled or intranasal) vaccination can effectively induce mucosal immunity, providing superior protection against infection compared to intramuscular injection [34]. To date, several inhaled or intranasal COVID-19 vaccines have been authorized, including DelNS1-2019-nCoV-RBD-OPT1, Convidecia Air, and iNCOVACC. While multiple approaches are being explored, the rational design of vaccine antigens and epitopes remains the core of achieving broad protection [35].

In our previous work [36], we designed a monomeric vaccine antigen, STFK_1628X_, which contains the S protein backbone of the Gamma variant (P.1 lineage) engineered to incorporate key RBD mutation sites characteristic of the Delta variant (B.1.617.2 lineage), together with the N-terminal domain derived from the B.1.620 lineage. STFK_1628X_ was subsequently combined with STFK_prototype_ based on the Wuhan-Hu-1 ancestral strain (GenBank: MN908947.3) to construct a bivalent vaccine, which was initially formulated with FH002C adjuvant. This bivalent vaccine demonstrated broad neutralizing activity against 19 circulating SARS-CoV-2 strains in hamsters and conferred protection against challenge with multiple variants, while also preventing transmission between cohoused hamsters, thereby showing promising broad-spectrum efficacy in preliminary proof-of-concept studies. To improve the safety profile of the bivalent vaccine and facilitate its further clinical application, the adjuvant was replaced with the widely used aluminum adjuvant. The resulting bivalent vaccine, composed of STFK_1628X_ and STFK_prototype_ and designated STFKB, was subsequently subjected to a series of preclinical studies. We comprehensively assessed the immunogenicity and safety of STFKB in mice, rats, and non-human primates. Its protective efficacy was further evaluated in a hamster challenge model using the Omicron BA.1 and BA.5 variants. Collectively, this study provides a systematic preclinical evaluation of a rationally designed broadly protective COVID-19 vaccine candidate and highlights its potential to address the challenges posed by antigenically diverse SARS-CoV-2 variants.

## 2. Materials and Methods

### 2.1. Animal Experiments and Ethical Statement

Female BALB/c mice (6–8 weeks old) were obtained from Shanghai SLAC Laboratory Animal Co., Ltd. (Shanghai, China). Male and female Sprague–Dawley rats (6–7 weeks old) were sourced from Zhejiang Vital River Laboratory Animal Technology Co., Ltd. (Hangzhou, China). Male and female guinea pigs (6 weeks old) were purchased from Qingdao Kangda Aibo Biotechnology Co., Ltd. (Qingdao, China). Mice, rats, and guinea pigs were housed under specific pathogen-free (SPF) conditions. Male and female rhesus monkeys (2–3 years old) were obtained from Guangxi Xiongsen Primate Experimental Animal Breeding Development Co., Ltd. (Pingnan, China, license number 0002977). Lakeview Golden (LVG) Syrian hamsters (6–8 weeks old) were acquired from Charles River Laboratories (Beijing, China). All animal experiments mentioned above were conducted in accordance with the Guide for the Care and Use of Laboratory Animals and approved by the Institutional Animal Care and Use Committee of Xiamen University (approval number: XMULAC20220030).

### 2.2. Cell Lines and Virus

BHK21-hACE2 cells, derived from BHK21 cells and stably overexpressing the human ACE2 receptor, were cultured in Dulbecco’s modified Eagle medium (DMEM; Sigma-Aldrich, St. Louis, MO, USA, D6429) supplemented with 10% fetal bovine serum (FBS; Gibco, Waltham, MA, USA, 10270106) and 1% penicillin-streptomycin (BasalMedia, Shanghai, China, S110JV).

In this study, the authentic viruses included the SARS-CoV-2 BA.1 variant (hCoV-19/China/AP309/2021, EPI_ISL_9005509) and the SARS-CoV-2 BA.5 variant.

### 2.3. SDS-PAGE, Native-PAGE, Western Blot and HPLC-SEC

SDS-PAGE analysis was performed using 4–12% precast gels (GenScript, Nanjing, China, YoungPAGE). Samples were mixed with reducing loading buffer (laboratory-prepared) and heated at 100 °C for 10 min before electrophoresis. A total of 5 μg of protein was loaded per lane. Electrophoresis was carried out in MOPS running buffer (GenScript) at 120 V for 1 h, followed by staining and destaining using the eStain^®^ L1 Protein Staining System (GenScript). For Native-PAGE, samples were mixed with Native Sample Buffer (Bio-Rad, Hercules, CA, USA) and loaded at 10 μg per lane onto precast gels from Thermo Fisher Scientific (Waltham, MA, USA). Electrophoresis was performed in Native-PAGE running buffer (Bio-Rad) at 80 V for 150 min, followed by staining and destaining using the eStain^®^ L1 Protein Staining System (GenScript). Western blotting was performed using the same sample preparation and electrophoresis conditions as SDS-PAGE, with 1 μg of protein loaded per lane. Following electrophoresis, proteins were transferred to a membrane using a wet transfer system (Bio-Rad). The membrane was washed with deionized water and blocked overnight with 5% skim milk prepared in 20 mM PBS (pH 7.4). After washing again with deionized water, the membrane was incubated for 1 h at room temperature with 36H6 [36] (a mouse anti-S protein monoclonal antibody selected in our laboratory and validated for WB detection), diluted in TBST containing 5% BSA. The membrane was then washed with PBST and incubated for 30 min at room temperature with an HRP-conjugated goat anti-mouse secondary antibody (Thermo Fisher Scientific, 31430, 0.16 μg/mL). After additional washes with PBST, the blot was developed using an enhanced chemiluminescence (ECL) detection kit (NCM Biotech, Suzhou, China). Protein bands were visualized using the FUSION FX7 Spectra multispectral imaging system (Vilber, Collégien, France).

Size-exclusion liquid chromatography (SEC) analysis of the two components of STFKB was performed using a Waters Alliance HPLC system equipped with a TSKgel G3000PWXL column. The column was equilibrated for 2 h with 20 mM phosphate-buffered saline (PBS) as the mobile phase at a flow rate of 1 mL/min. Subsequently, 20 μL of protein sample was injected, and elution was carried out with the same mobile phase for 30 min.

### 2.4. Vaccine Preparation

The STFKB vaccine and its corresponding adjuvant solution were manufactured by Wantai BioPharm (Beijing, China). The STFKB vaccine was produced using the ExpiCHO expression system (Thermo Fisher Scientific) and purified by Q-FF Sepharose ion-exchange chromatography (Cytiva, Marlborough, MA, USA). The negative control consisted of 0.9% sodium chloride injection solution. In the animal immunization experiments, the STFKB vaccine (antigen concentration: 120 µg/mL; Alum concentration: 840 μg/mL) was used. The positive control for guinea pigs was ovalbumin (Aladdin^®^, Shanghai, China, 4 mg/mL).

### 2.5. Mouse Immunization

Mice (*n* = 6) were immunized by the intramuscular route (i.m.) once every three weeks for a total of three doses. The vaccine was administered intramuscularly into the thigh muscles of both hind limbs using a 1 mL syringe. Doses (150 µL/animal) contained 0.1, 1, 10, or 20 µg antigen. Blood samples were collected from the orbital sinus for two weeks after each immunization.

### 2.6. Rat Immunization

Rats (*n* = 10) were immunized by the intramuscular route (i.m.) once every two weeks for a total of four doses. The vaccine was administered intramuscularly into the thigh muscles of both hind limbs. The low-dose and high-dose groups received 60 µg (0.5 mL) and 120 µg (1 mL) per animal, corresponding to 1-fold and 2-fold the clinical dose, respectively. To fully assess toxicity exposure, the dosing frequency was increased by one additional administration (N + 1) compared to the maximum anticipated clinical dosing frequency, and the dosing interval was shorter than the proposed clinical dosing interval (The proposed clinical immunization interval is 3 weeks). Blood samples were collected from the jugular vein on D13, D27, D41, D56, and D71. Body temperatures were measured rectally using an MC-347 electronic thermometer.

### 2.7. Rhesus Monkey Immunization

Rhesus monkeys (one male and one female in the control group, and three males and three females in the other groups) were immunized by the intramuscular route (i.m.) once every three weeks for a total of three doses. The vaccine was administered intramuscularly into the lateral thigh muscle. The low-dose and high-dose groups received 6 μg (0.5 mL) and 60 μg (0.5 mL) of vaccine, respectively. Blood samples were collected from a peripheral vein of the limb or the femoral vein on D14, D35, and D56.

### 2.8. Guinea Pig Immunization

Guinea pigs (*n* = 6) were sensitized by the rapid intramuscular injection every other day on D0, D2, and D4, for a total of three doses (the low-dose and high-dose groups received 60 µg and 120 µg per animal, respectively). Both the low-dose group and the positive control group received 0.5 mL per animal, while other groups received 1 mL per animal. The provocation test was administered via rapid dorsal foot vein injection on the 14th day after the last sensitization dose (D18). For the provocation tests, each group included one female and two males, while dosage and volume were double those of the sensitization tests (the low-dose and high-dose groups received 120 μg and 240 μg per animal, respectively).

### 2.9. Hamster Immunization

In the challenge protection experiment, hamsters (*n* = 8) were immunized by the intramuscular route (i.m.) once every three weeks for a total of three doses (10 µg in 200 µL per animal). The vaccine was administered intramuscularly into the thigh muscles of both hind limbs. The challenge occurred two weeks after the last immunization.

### 2.10. Pseudovirus (PsV) Neutralization Assay

SARS-CoV-2 neutralizing antibodies were quantified using a VSV-based pseudovirus (PsV) system [37]. The specific experimental steps were as follows: BHK21-hACE2 cells (3 × 10^4^ cells/well) were seeded in 96-well plates 12 h before the assay and reached 70–80% confluency at the time of infection. Test sera were heat-inactivated at 56 °C for 30 min and serially diluted in U-bottom 96-well plates. Briefly, 5 μL of inactivated serum was mixed with 195 μL of DMEM containing 10% FBS to obtain a 40-fold initial dilution, followed by three-fold serial dilutions by transferring 60 μL of the previous dilution into 120 μL of DMEM containing 10% FBS. A total of eight or nine serial dilutions were prepared. Each plate included eight wells without test sera as the MOCK control and eight wells with three-fold serial dilutions of the nAb SA55 [38], which was based on a publicly available sequence and produced in our laboratory according to the corresponding patent application, as the positive control. Then, 80 μL of each serum dilution was mixed with 20 μL of diluted SARS-CoV-2 pseudovirus, resulting in a final serum dilution of 1:50. After incubation at 37 °C for 1 h, 80 μL of the mixture was added to the cell plates. After incubation at 37 °C with 5% CO_2_ for 16 h, GFP fluorescence was imaged using an EnSight multimode plate reader (PerkinElmer, Waltham, MA, USA), and GFP-positive cells in each well were quantified using the accompanying software.

### 2.11. Anti-STFK IgG, Anti-STFKx IgG and Anti-Spike IgG Measurement

The IgG antibody titers against the STFK_prototype_, STFK_1628X_ and Spike proteins were determined using the enzyme-linked immunosorbent assay (ELISA). Microplates precoated with recombinant antigens of STFK_prototype_ or STFK_1628X_ were provided by Wantai BioPharm. The Spike proteins corresponding to different circulating variants were expressed in our laboratory using the ExpiCHO expression system (Thermo Fisher Scientific, Waltham, MA, USA) and subsequently coated onto microplates for the ELISA. For the ELISA assay, each well was coated with 200 ng of protein. Prior to sample loading, plates were blocked with 5% skim milk prepared in 20 mM PBS (pH 7.4) to eliminate non-specific binding. Test samples were serially diluted starting at 1:100 in 20% newborn bovine serum (NBS) diluted in 20 mM PBS (pH 7.4) and added to the pre-coated wells, followed by incubation for 30 ± 5 min. After incubation, plate washing was performed using an automatic plate washer (DEM-3, Topu, Beijing, China) with PBST buffer (20 mM PBS, pH 7.4, containing 0.05% Tween-20) to remove unbound components. Subsequently, species-specific HRP-conjugated secondary antibodies were added for further incubation for 30 ± 5 min: goat anti-rat IgG (Proteintech, Rosemont, IL, USA, SA00001-15) for rat samples and goat anti-monkey IgG (Abcam, Cambridge, UK, ab112767) for monkey samples. The chromogenic substrate (Wantai BioPharm, Beijing, China) and stop buffer (Wantai BioPharm, Beijing, China) were used for color development and reaction termination. The absorbance value of each well was measured at 450 nm and 630 nm using a microplate reader (PHOMO, Autobio Diagnostics, Zhengzhou, China). All samples were detected in replicate wells to ensure data reliability.

### 2.12. Biochemical Markers and Immune Function Measurement

Blood samples were collected into sterile tubes and allowed to clot naturally to obtain serum. Following centrifugation at 1500× *g* for 10 min, serum was analyzed for biochemical markers using a Hitachi-7180 automated analyzer.

Immunoglobulins and complement components, including IgM, IgG, IgA, C3, and C4, were measured in serum using a Hitachi-7180 automated analyzer.

### 2.13. SARS-CoV-2 Virus Challenges in Hamster

For the challenge protection experiments, hamsters were anesthetized two weeks post-immunization and challenged intranasally with authentic viruses: BA.1 (1 × 10^5^ PFU) and BA.5 (1 × 10^4^ PFU). All challenge doses were diluted in PBS to 100 µL (50 µL per nostril) and kept on ice until administration. Non-immunized hamsters served as the control group. Animals were monitored daily for weight changes and humanely euthanized at 5 days post-challenge. Finally, lung, nasal turbinate, and trachea tissues were harvested for viral RNA quantification, and gross lung pathology was observed. All experiments were conducted in the Animal Biosafety Level 3 (ABSL-3) facility.

### 2.14. SARS-CoV-2 RNA Quantification

Viral RNA quantification was performed on homogenized tissues (lungs, 2 pieces; half nasal turbinate; trachea) collected in PBS-containing tubes using a TissueLyser II (Qiagen, Hilden, Germany). The viral RNA was extracted using the QIAamp Viral RNA Mini Kit (Qiagen), followed by qRT-PCR experiments with the SARS-CoV-2 RT-PCR Kit (Wantai BioPharm, Beijing, China, WS-1248) (primers and probes targeting the virus ORF1ab) with a Bio-Rad CFX96 Touch Real-Time PCR Detection System.

### 2.15. Quantification and Statistical Analysis

In the experiment, the sample number, vaccine dose and virus dose were described in the figure and the Materials and Methods. Each experiment was performed in three technical replicates, and the data shown are representative results from one independent experiment. Mann–Whitney test and One-way ANOVA Multiple comparisons-Dunnett’s multiple comparisons test were used to analyze single-factor comparisons. Two-way ANOVA Multiple comparisons-Dunnett’s multiple comparisons test, Tukey’s multiple comparisons test and Šídák’s multiple comparisons test were used to analyze dual-factor variables. Adjusted *p*-values were indicated as follows: *p* < 0.0001 as ****, *p* < 0.001 as ***, *p* < 0.01 as **, *p* < 0.05 as *, and no significant difference as ns. Statistical analyses were performed using GraphPad Prism 9.5.1 or GraphPad Prism 10.1.2.

## 3. Results

### 3.1. STFKB Induces STFK- and STFK_1628X_-Specific Antibodies and Broadly Neutralizing Responses in Animal Models

**Figure 1:**
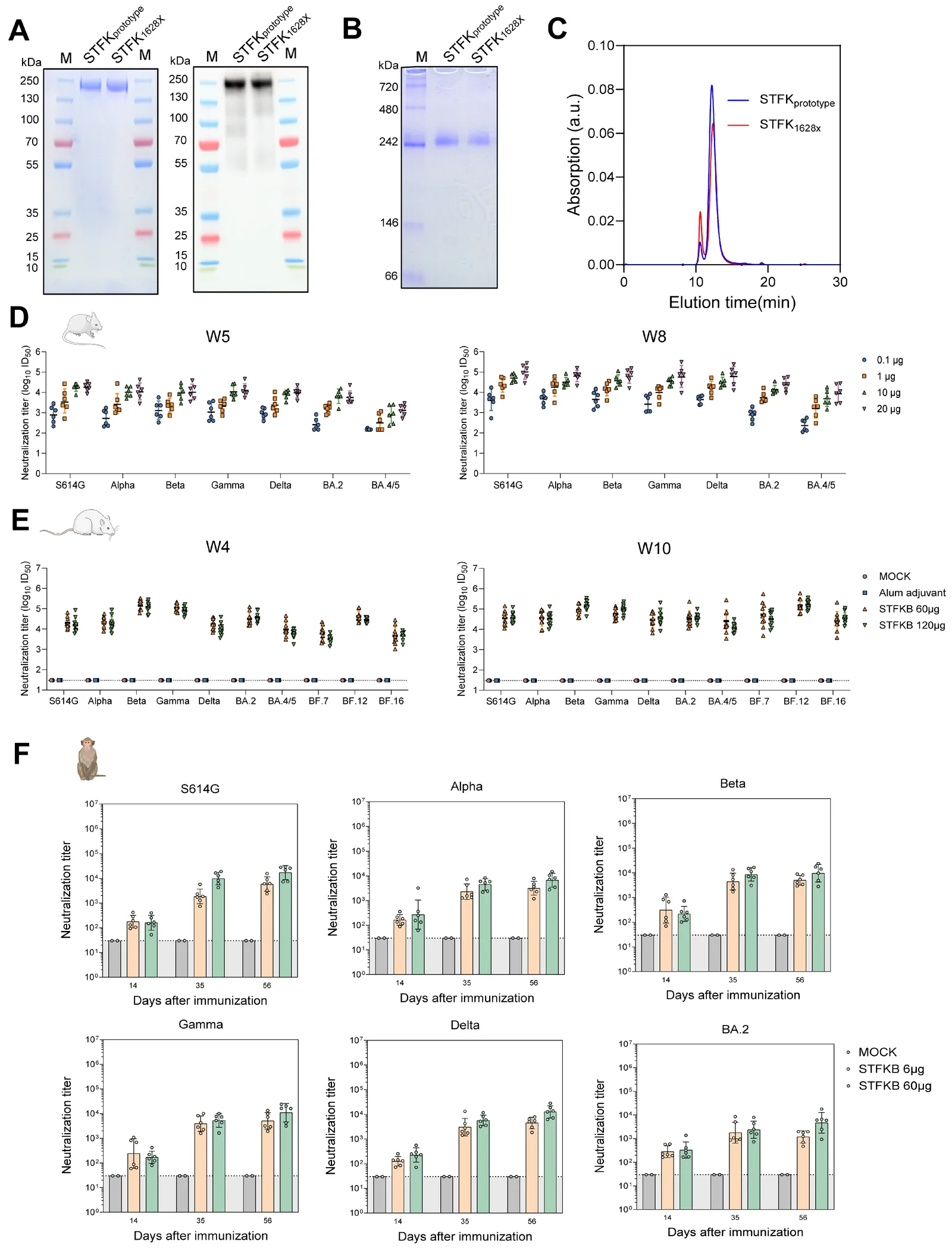
STFKB elicits significant neutralizing antibody responses in animal models. (**A**) SDS-PAGE identification of the two components of the bivalent vaccine. Loading amount: 5 μg per lane. Western blot (WB) identification of the two components of the bivalent vaccine using Anti-S protein antibody. Loading amount: 1 μg per lane. (**B**) Native PAGE analysis of the bivalent vaccine components. Loading amount: 10 μg per lane. (**C**) HPLC-SEC analysis of the bivalent vaccine components. (**D**) The neutralization antibody titer of sera from mice (*n* = 6) vaccinated with bivalent STFKB after two doses or three doses. (**E**) The neutralization antibody titer of sera from rats (*n* = 10) vaccinated with bivalent STFKB after two doses or four weeks into the recovery period. All the rats received four vaccinations (administered on weeks 0, 2, 4, and 6), with the negative control group receiving 0.9% sodium chloride solution and the adjuvant control group receiving Alum adjuvant. (**F**) The neutralization antibody titer of sera from rhesus monkeys (*n* = 6) vaccinated with bivalent STFKB. All the rhesus monkeys received three vaccinations (administered on weeks 0, 3 and 6), with the negative control group (*n* = 2) receiving 0.9% sodium chloride solution. The time points shown in the figures (**D**,**E**) indicate the weeks after the primary immunization. The dashed line represents the lower limit of detection (**E**,**F**). The animal figures were used from pictures provided by Servier Medical Art (https://smart.servier.com/, accessed on 26 January 2025), provided by Servier, licensed under a Creative Commons Attribution 4.0 unported license.

The batch-produced STFK_prototype_ and STFK_1628X_ proteins were first characterized. SDS-PAGE was used to assess the purity of the vaccine components, Western blotting confirmed their identity, and native PAGE and HPLC-SEC indicated that the proteins existed predominantly in monomeric form (Figure 1A–C). Although Western blotting and HPLC-SEC detected minor heterogeneous species generated during preparation, the target antigen remained the major component of the preparation, supporting its use for further vaccine evaluation. Subsequently, the two monomeric proteins were combined and formulated with aluminum adjuvant to generate the bivalent vaccine STFKB, which was then used for animal immunization.

We evaluated the immunogenicity of STFKB in BALB/c mice. Mice were immunized with three doses of STFKB at three-week intervals, and sera were collected after immunization for antibody analysis. STFKB induced robust neutralizing antibody responses after two doses, with further enhancement after the third dose across the tested SARS-CoV-2 variants (Figure 1D). These data indicate that STFKB can rapidly elicit variant-reactive humoral immunity in mice in a dose-dependent manner.

We subsequently assessed STFKB immunogenicity in rats using a four-dose schedule (N + 1, relative to the proposed clinical frequency) administered at two-week intervals, with doses of 60 µg and 120 µg, representing 1-fold and 2-fold the proposed clinical doses, respectively. Control groups included mock-immunized and adjuvant-only animals. Humoral immune responses were evaluated by measuring neutralizing antibodies against multiple variants, as well as anti-STFK IgG and anti-STFK_1628x_ IgG (hereafter referred to as anti-STFKx IgG). Consistent with observations in mice, STFKB induced high-titer neutralizing antibodies against a broad panel of variants in rats. After two doses, GMTs against BA.4/5 were 9572 in the 60 µg group and 5967 in the 120 µg group. Substantial neutralizing activity was also observed against other Omicron sublineages, including BF.7 (GMTs: 6108 and 3239 for 60 µg and 120 µg, respectively), BF.12 (GMTs: 42,319 and 27,581), and BF.16 (GMTs: 4627 and 4734) (Figure 1E). Following the third dose, neutralizing titers further increased, with antibody GMTs against BA.4/5 reaching 9974 and 12,521 for 60 µg and 120 µg, respectively. Remarkably, GMTs against BF.12 reached GMTs of 221,755 in the 60 µg group and 105,394 in the 120 µg group (Figure S1A). Neutralizing antibody titers in the high-dose group did not exceed those of the low-dose group, showing comparable or slightly lower values. This suggests that the 60 µg dose may have already saturated the immune response in rats, where additional antigen escalation did not yield a proportional increase in antibody production. During the recovery phase (the observation period after the final immunization), the differences in neutralizing antibody titers among different variants decreased, and the titers remained at appreciable levels across all variants (Figure 1E). Anti-STFK and anti-STFKx IgG responses were below the limit of detection (<100) in both control groups throughout the study. In vaccinated animals, high antibody titers were detectable as early as two weeks post-first dose, peaked following the second immunization, and remained elevated until four weeks after the recovery period (Figure S1B).

To further evaluate translational potential, we assessed STFKB immunogenicity in non-human primates using rhesus macaques. A single dose of STFKB rapidly induced neutralizing antibodies against all tested strains, whereas titers in control groups were below 30. Neutralizing titers increased substantially following the second dose (serum samples collected at Day 35). STFKB elicited high neutralizing titers against both the ancestral S614G strain and the Omicron BA.2 variant. In the 6 µg group, GMTs reached 1856 against S614G and 1790 against BA.2; in the 60 µg group, titers were substantially higher, at 9752 and 2373, respectively. Higher antigen doses were consistently associated with more robust neutralizing responses. Following the third dose, titers increased further, with GMTs against S614G reaching 5845 in the 6 µg group and 17,072 in the 60 µg group, and GMTs against BA.2 reaching 1187 and 4615, respectively (Figure 1F). STFKB also induced robust IgG responses in macaques. As observed for neutralizing antibodies, a single dose elicited anti-STFK IgG and anti-STFKx IgG, while control animals remained negative (<100). Anti-STFK IgG titers peaked after two doses, attaining GMTs of 650,199 in the 6 µg group and 1,158,524 in the 60 µg group, and remained elevated at two weeks following the third dose (459,760 and 1,158,524, respectively). Anti-STFKx IgG responses followed similar kinetics, peaking after two doses and remaining at a detectable level throughout the study period (Figure S1C). To further assess humoral immune responses against more recently circulating variants, sera from rhesus macaques after three immunizations were tested against NB.1.8.1, XFG, KP.3, JN.1, and XBB.1.5. Neutralizing antibody GMTs against NB.1.8.1, XFG, and KP.3 ranged from 32 to 42, while those against JN.1 were 42 in the 6 μg group and 82 in the 60 μg group. Anti-Spike IgG titers against XBB.1.5, NB.1.8.1, and XFG were all above 10^5. These data indicate detectable neutralizing activity against more recently circulating variants, together with strong binding antibody responses after vaccination (Figure S1D,E).

### 3.2. STFKB Is Well Tolerated in Rodents, Guinea Pigs, and Rhesus Macaques

**Figure 2:**
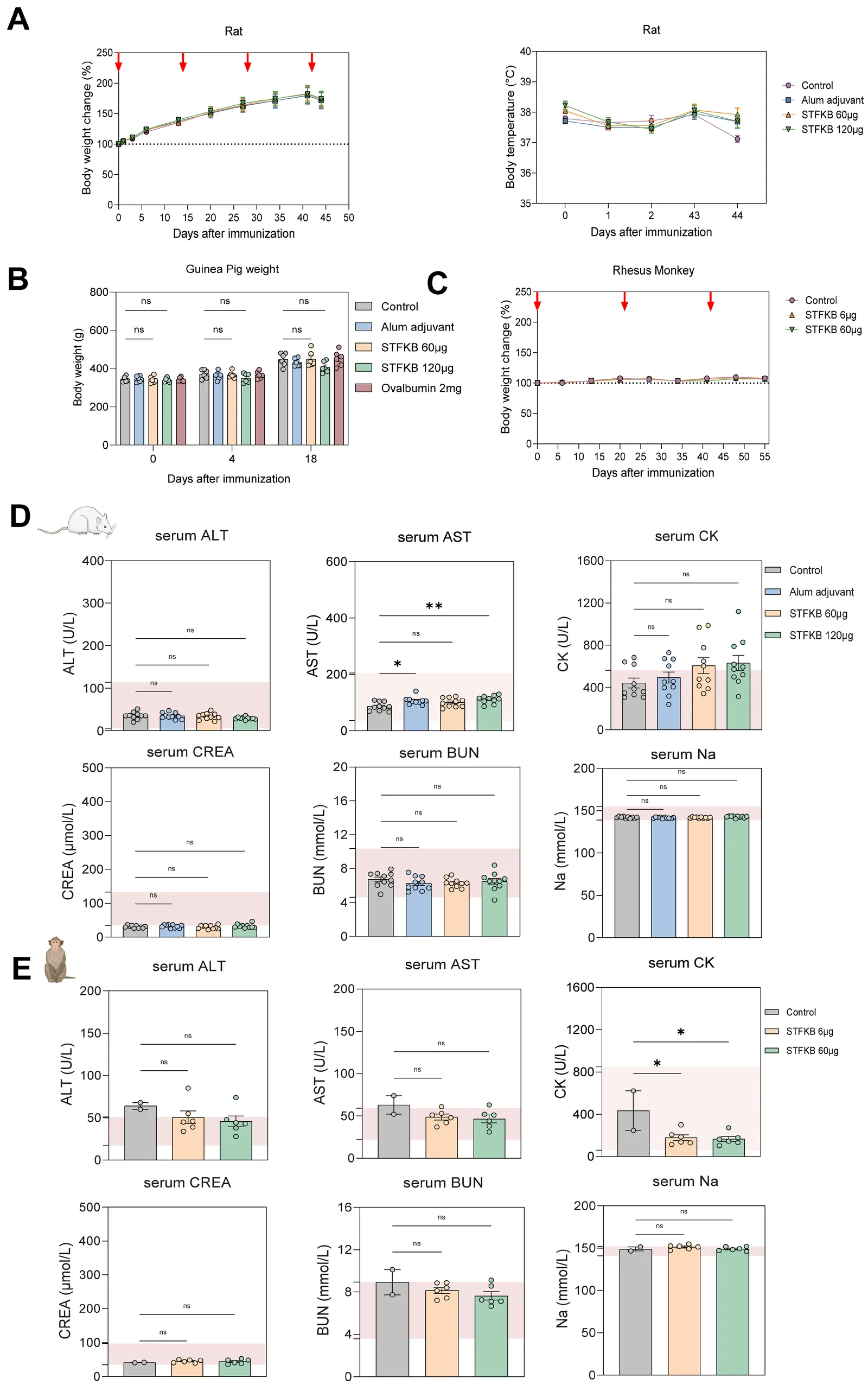
STFKB demonstrates a good safety profile in animal models. (**A**) The curves represent the body weight change or the body temperature of rats vaccinated with bivalent STFKB. The control groups were also established. Data were mean ± SEM. The red arrows indicated immunization time points. (**B**) The body weight of guinea pigs (*n* = 6) in the bivalent STFKB active systemic anaphylaxis study. The sensitization route of administration was intramuscular injection (administered on days 0, 2 and 4) for a total of three times. Fourteen days after the last sensitization (D18), the challenge was administered through dorsal vein injection in the foot, using twice the sensitization dose of bivalent STFKB. The experiment established a negative control group (0.9% sodium chloride injection), an adjuvant control group (Alum adjuvant) and a positive control group (ovalbumin). (**C**) The curves represented the body weight change in rhesus monkeys vaccinated with bivalent STFKB. The control groups were also established. Data were mean ± SEM. The red arrows indicated immunization time points. (**D**) Biomarkers of hepatic (ALT, AST), cardiac enzyme (CK), renal (CREA, BUN), and electrolyte (Na) in SD rats vaccinated with bivalent STFKB after four doses were shown. Data were mean ± SEM. The pink shaded area represented the reference range (the above reference ranges were sourced from the Division of Laboratory Animal Medicine, David Geffen School of Medicine at UCLA: https://labs.dgsom.ucla.edu/dlam/diagnostic/, accessed on 26 January 2025). (**E**) Biomarkers of hepatic (ALT, AST), cardiac enzyme (CK), renal (CREA, BUN), and electrolyte (Na) in rhesus monkeys vaccinated with bivalent STFKB after three doses were shown. Data were mean ± SEM. The pink shaded area represented the reference range (the above reference ranges were sourced from the Division of Laboratory Animal Medicine, David Geffen School of Medicine at UCLA: https://labs.dgsom.ucla.edu/dlam/diagnostic/, accessed on 26 January 2025). For intergroup statistical comparisons, Tukey’s multiple comparisons test (**B**) and Dunnett’s multiple comparisons test (**D**,**E**) were used. Asterisks demonstrated statistical significance (** *p* < 0.01, * *p* < 0.05, ns indicated not significant). The animal figures were used from pictures provided by Servier Medical Art (https://smart.servier.com/, accessed on 26 January 2025), provided by Servier, licensed under a Creative Commons Attribution 4.0 unported license.

To comprehensively assess the safety profile of STFKB and support its potential for clinical translation, we evaluated multiple safety parameters across three species of animals. In rats, no significant weight loss or abnormal body temperature fluctuations were observed during the four-dose immunization regimen when compared with mock-immunized and adjuvant-only control groups (Figure 2A). These findings indicate that STFKB administration was well tolerated locally and systemically in rodents.

We further evaluated potential allergic responses in guinea pigs using a systemic active anaphylaxis model. Guinea pigs were included as one of the animal models for vaccine safety evaluation in this study. This species is commonly used in vaccine safety testing in China because of its sensitivity to immune responses, allergic reactions, and local irritancy, making it suitable for detecting both local and systemic vaccine-induced allergic reactions. Animals received three intramuscular sensitizing doses of STFKB, followed by a 14-day incubation period and subsequent intravenously administered double-dose provocation. Throughout the sensitization phase and following provocation, no fatalities, near-death events, or anaphylactic symptoms were observed in STFKB-immunized animals. Moreover, body weight trajectories remained comparable to those of control groups throughout the observation period, demonstrating the absence of vaccine-related hypersensitivity reactions (Figure 2B).

In non-human primates, rhesus macaques immunized with three doses of STFKB showed no significant weight reduction compared to control animals, further supporting the favorable tolerability of the vaccine candidate across species (Figure 2C).

To evaluate potential organ-specific toxicity, we analyzed serum biochemical markers in rats following completion of the four-dose immunization regimen. No statistically significant differences were observed between STFKB-immunized animals and mock controls for key hepatic (alanine aminotransferase [ALT]), cardiac (creatine kinase [CK]), renal (creatinine [CREA], blood urea nitrogen [BUN]), or electrolyte (sodium [Na]) parameters (Figure 2D). A slight, transient elevation in aspartate aminotransferase (AST) was noted in some vaccinated animals; however, this finding was attributed to the presence of the aluminum adjuvant rather than the bivalent STFKB antigen itself.

In rhesus macaques receiving three doses of STFKB, comprehensive serum biochemistry analysis revealed no significant differences between vaccinated and control groups for hepatic (ALT, AST), renal (CREA, BUN), or electrolyte (Na) markers (Figure 2E). An isolated elevation of CK (Monkey #111: CK = 622 U/L) was observed in the mock control group, which comprised only two animals; this single outlier accounted for the elevated mean CK value in the control group and was determined to be unrelated to vaccine administration based on the absence of similar findings in vaccinated animals and the lack of clinical correlates [39]. Analysis of additional serum biomarkers and immunological parameters demonstrated no vaccine-associated abnormalities across any of the species evaluated (Table S1).

Collectively, these safety assessments across rodent and non-human-primate models indicate that STFKB was well tolerated under the tested preclinical conditions and support its further development. Because the non-human-primate control group was limited in size and the follow-up period was finite, these findings should be interpreted as preclinical tolerability evidence rather than definitive clinical safety evidence.

### 3.3. STFKB Protects Hamsters Against Omicron BA.1 and BA.5 Challenge

To evaluate the protective efficacy of STFKB against early Omicron variants, we utilized a golden Syrian hamster challenge model. Animals were immunized with three doses of STFKB administered at three-week intervals, followed by intranasal challenge with either Omicron BA.1 virus (1 × 10^5^ PFU) or Omicron BA.5 virus (1 × 10^4^ PFU). Protective efficacy was assessed through multiple parameters, including post-challenge body weight changes, viral RNA loads in respiratory tissues (lungs, nasal turbinates, and trachea), and gross lung pathology.

**Figure 3:**
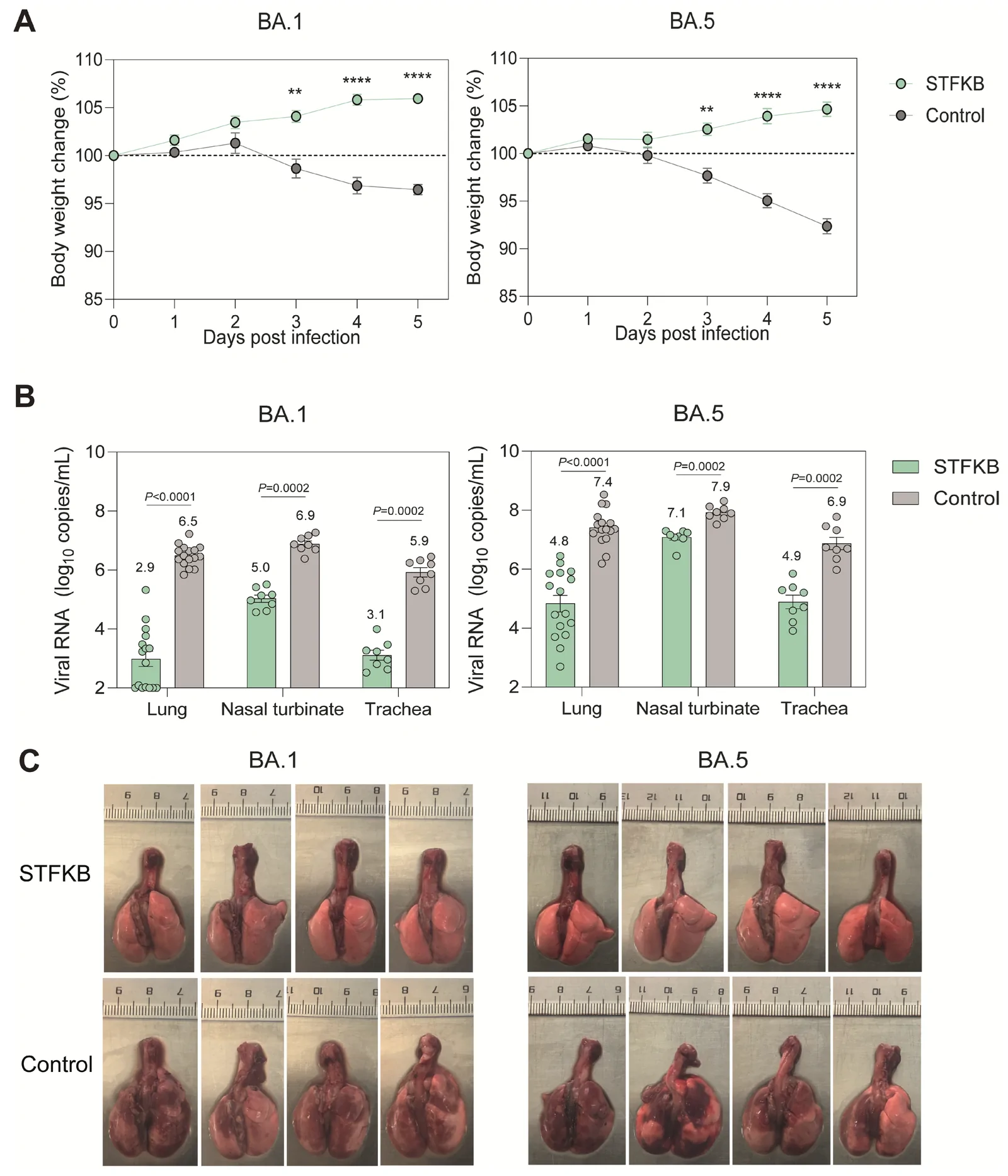
Protective efficacy of the bivalent vaccine STFKB against Omicron BA.1 and BA.5 challenge in hamsters. (**A**) The body weight changes in hamsters (*n* = 8) immunized with three doses of bivalent vaccine STFKB (10 µg per animal) after BA.1 or BA.5 variant challenge were shown. Data are mean ± SEM. (**B**) The viral RNA loads (targeting the ORF1ab region) of the lung tissue, nasal turbinate and trachea from hamsters were quantified. (**C**) Gross observation of the lung tissues from hamsters immunized with three doses of bivalent vaccine STFKB on the fifth day after BA.1 or BA.5 variant challenge. For intergroup statistical comparisons, Šídák‘s multiple comparisons test (**A**) and Mann–Whitney test (**B**) were used. Asterisks demonstrated statistical significance (**** *p* < 0.0001, ** *p* < 0.01).

Following challenge with Omicron BA.1 virus, hamsters immunized with STFKB maintained body weight, with a mean body weight increase of +5.9% at 5 days post-infection (dpi). In striking contrast, control animals demonstrated progressive weight loss beginning at 3 dpi, reaching a mean decline of −3.6% by 5 dpi. A similar protective pattern was observed following BA.5 virus challenge: vaccinated animals maintained stable weight with a mean increase of +4.6% at 5 dpi, whereas control animals experienced significant and progressive weight loss, averaging −7.7% at 5 dpi. Statistically significant differences in weight trajectories between vaccinated and control groups emerged as early as 3 dpi for both variant challenges, underscoring the rapid protective effect conferred by STFKB immunization (Figure 3A).

Viral RNA loads in respiratory tissues were quantified at 5 dpi to assess the capacity of STFKB to restrict viral replication and dissemination. In the BA.1 challenge model, lung viral RNA levels in vaccinated hamsters remained below 3 log_10_ copies/mL, whereas control animals exhibited viral loads of 6.5 log_10_ copies/mL, representing a reduction of greater than 3 log_10_. In nasal turbinates, vaccinated animals showed viral loads of approximately 5.0 log_10_ copies/mL, while tracheal loads were approximately 3.1 log_10_ copies/mL, both substantially reduced compared to control animals. Similarly, in hamsters challenged with Omicron BA.5 virus, STFKB-immunized animals demonstrated reductions of 0.8 to 2.6 log_10_ in viral RNA loads across all respiratory tissues examined relative to unvaccinated controls (Figure 3B). These data demonstrate that STFKB immunization effectively limits viral replication in both upper and lower respiratory tracts against antigenically distinct Omicron sublineages.

Gross pathological examination of lung tissues further corroborated the protective efficacy of STFKB. Following challenge with either Omicron BA.1 or BA.5 virus, lungs harvested from vaccinated hamsters retained normal morphological appearance, characterized by healthy pink coloration, absence of hemorrhagic foci, and no evidence of significant size alteration or consolidation. In marked contrast, lungs from control animals challenged with either variant exhibited extensive hemorrhage, widespread tissue necrosis, and pathological changes indicative of severe pulmonary damage and acute respiratory distress (Figure 3C). These macroscopic findings are consistent with the weight stability and reduced viral loads observed in vaccinated animals, collectively demonstrating that STFKB immunization confers effective protection against pulmonary pathology induced by divergent Omicron sublineages.

Taken together, these challenge studies in the golden Syrian hamster model provide compelling evidence for the protective efficacy of STFKB against early Omicron variants. The vaccine candidate effectively prevented morbidity as measured by weight loss, significantly reduced viral replication in both upper and lower respiratory tracts, and preserved normal lung architecture following challenge with antigenically distinct Omicron sublineages BA.1 and BA.5. These findings support the potential of STFKB as a broad-spectrum vaccine candidate capable of conferring protection against early Omicron variants of concern.

## 4. Discussion

In this study, we employed the previously engineered STFK_1628X_—a next-generation immunogen incorporating key variant mutations from epidemiologically significant variants—and combined it with the ancestral strain antigen (STFK_prototype_) to generate the bivalent vaccine candidate STFKB [36]. Comprehensive preclinical assessment demonstrated robust immunogenicity across multiple species. In murine models, STFKB elicited high-titer neutralizing antibodies against all tested variants, with titers remaining detectable at 1 × 10^2^ to 1 × 10^4^ against Omicron sublineages BA.2 and BA.4/5 following three-dose immunization. In rats, STFKB similarly induced strong immunogenicity, with neutralizing antibody titers against BA.2 comparable to those against the ancestral strain after two doses. During the four-week recovery period, all neutralizing antibody titers remained detectable at elevated levels, supporting the vaccine’s broad cross-neutralizing capacity. Future extended studies will be carried out to further demonstrate the durability of this response. In non-human primates, peak binding and neutralizing antibody titers were achieved following two doses of STFKB. This immunogenicity profile, coupled with broad reactivity against antigenically distinct variants of concern including BA.2 and BA.4/5, indicates substantial cross-protective potential. Although sera from rhesus macaques after three immunizations showed limited neutralizing activity against the more recently circulating variants, detectable cross-reactive binding responses and modest neutralizing activity were still observed. These findings suggest that the vaccine-induced humoral response was not entirely restricted to the early variants tested in the main study. At the same time, the limited neutralization level also highlights the need for further antigen optimization to improve coverage against more recently evolved variants (Figure 1 and Figure S1). Comprehensive safety evaluation across species revealed no significant abnormalities in body weight, temperature, or serum biomarkers, establishing a favorable preclinical safety profile for STFKB (Figure 2).

Furthermore, STFKB demonstrated excellent protective efficacy in hamster challenge models, with immunized animals showing no significant weight loss and minimal pulmonary pathology following challenge with either Omicron BA.1 or BA.5 variants, in marked contrast to unvaccinated controls that developed severe lung damage (Figure 3). Collectively, these findings demonstrate that STFKB exhibits favorable safety, potent and broad immunogenicity, and effective protection against diverse Omicron lineages in both upper and lower respiratory tracts, positioning it as a highly promising next-generation vaccine candidate. Our observations are consistent with emerging evidence from other studies [31,40], indicating that rationally designed bivalent vaccines can achieve broad-spectrum protective efficacy against antigenically diverse SARS-CoV-2 variants.

The design strategy of STFK_1628X_—employing antigenically divergent immunogens to broaden immune coverage while maintaining structural integrity—enables the bivalent formulation to effectively neutralize both ancestral and evolutionarily distant variant strains. The ancestral strain S protein was included to maximize complementarity with STFK_1628X_ and preserve conserved epitopes, thereby broadening cross-protective immune responses against diverse variants. Nevertheless, we acknowledge that the extent of protection against more recently circulating variants remains limited and may require further antigen optimization. By incorporating the Gamma variant backbone with key Delta variant RBD mutations and the B.1.620-derived NTD, STFK_1628X_ presents a broader repertoire of neutralizing epitopes to the immune system, potentially focusing responses toward conserved regions while also generating variant-specific antibodies. Building upon our prior research [36], the present study provides comprehensive preclinical evidence supporting the robust immunogenicity and favorable safety profile of STFKB. Given the accelerating pace of immune-evasive variant emergence, the development of such broad-spectrum vaccines represents an urgent priority for sustainable pandemic control. The bivalent COVID-19 vaccine we have designed demonstrates broad-spectrum efficacy in preclinical models, and the rational design approach employed herein may inform vaccine development strategies against other rapidly mutating pathogens with pandemic potential.

This study has several limitations that should be acknowledged. Firstly, our investigation focused primarily on humoral immune responses without comprehensive assessment of cellular immunity, thereby not fully capturing the complete landscape of vaccine-elicited immune protection. Given the established role of cellular immunity in viral clearance and disease attenuation, future studies should characterize T-cell responses elicited by STFKB and their contribution to protective efficacy. Secondly, another limitation of this study is that the protection experiments against BA.1 and BA.5 cannot be fully equated with protection against currently circulating variants. Although BA.1 and BA.5 remain representative Omicron lineages for evaluating cross-protective immunity, these findings cannot be directly extrapolated to the currently prevalent or future variants. Further studies will be conducted to assess vaccine protection against more recent circulating variants. Thirdly, while the non-human primate studies provided valuable translational data, the sample sizes were modest and may limit the generalizability of findings. Larger-scale studies in non-human primates, as well as eventual clinical trials in human populations, will be necessary to confirm the safety, immunogenicity, and protective efficacy observed in these preclinical models. Fourth, this study lacks direct comparison with currently approved vaccines, which will be addressed in future studies to better assess the application advantages of this vaccine candidate. Despite these limitations, the comprehensive preclinical data presented herein establish STFKB as a promising broad-spectrum vaccine candidate warranting further clinical development.

## Data Availability

All datasets generated or analyzed during this study are included in this published article and its Supplementary Information Files. Additional raw data supporting the findings are available from the corresponding author upon reasonable request.

## Supplementary Materials

The following supporting information can be downloaded at: https://www.mdpi.com/article/10.3390/v18090976/s1; Figure S1: Serum neutralizing antibody titers and STFK-specific, STFKx-specific, and Spike-specific IgG titers in rats and rhesus monkeys; Table S1: Physiological, biochemical, and immunological parameters of different animal models; Table S2: Amino acid sequences of the bivalent protein vaccine components.
