# Supplementary material for: An Engineered Spike Immunogen Drives Broad Protection of a Bivalent Recombinant Protein COVID-19 Vaccine in Animal Models
Source: Viruses. 2026 Sep 4;18(9):976. doi: 10.3390/v18090976 (PMC13611973; doi:10.3390/v18090976)
Supplement: Supplementary file 1 [file viruses-18-00976-s001.zip › Figure S1-Serum neutralizing antibody titers and STFK-specific, STFKx-specific, and Spike-specific IgG titers in rats and rhesus monkeys..pdf]

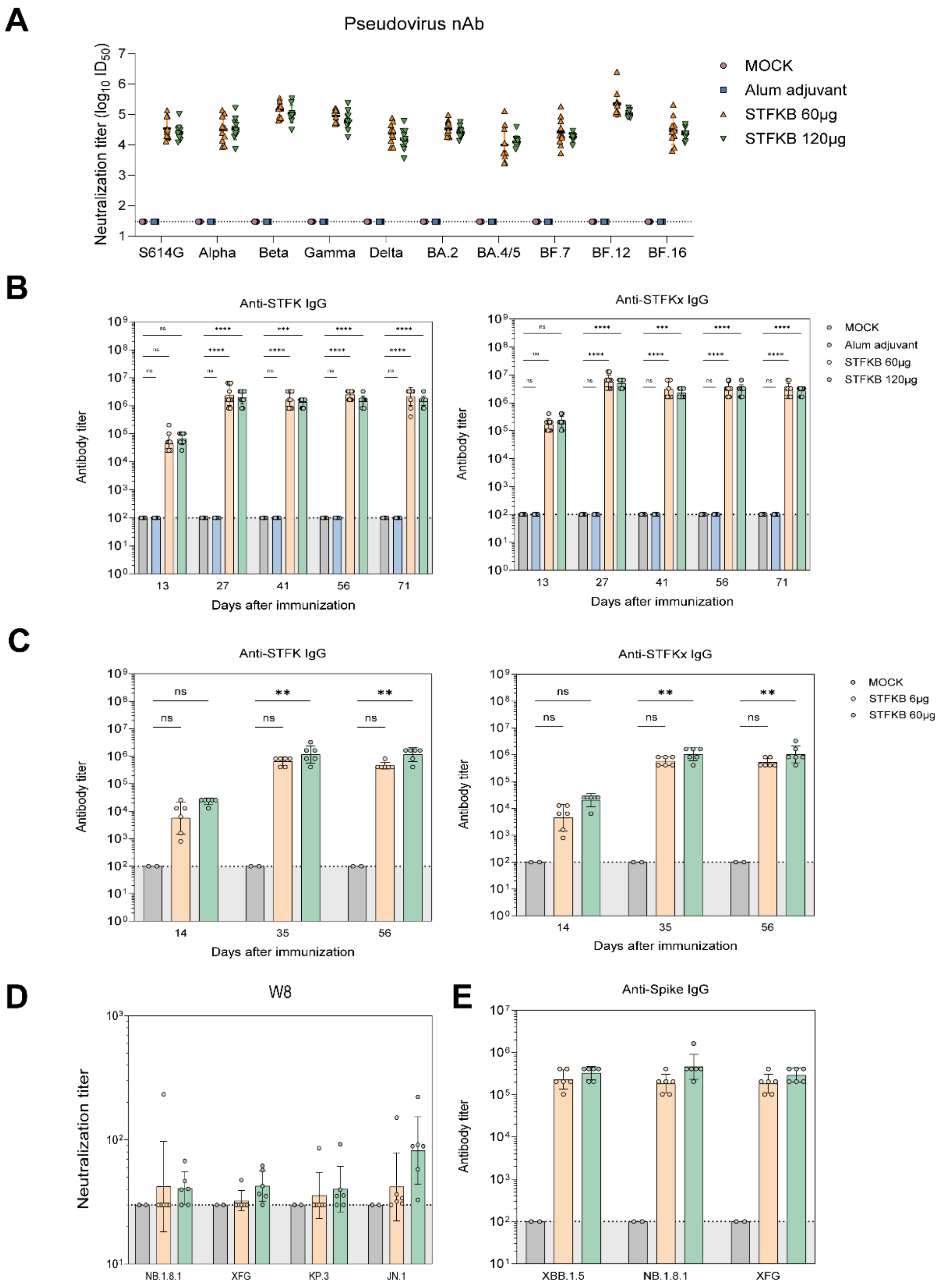


**Figure S1.** Serum neutralizing antibody titers and STFK-specific, STFKx-specific, and Spike-specific IgG titers in rats and rhesus monkeys.

(A) The neutralization antibody titer of sera from rats vaccinated with bivalent STFKB after three doses.

(B) The anti-STFK and anti-STFK$_{1628X}$ (anti-STFKx) IgG titer of sera from rats vaccinated with bivalent STFKB.

(C) The anti-STFK and anti-STFKx IgG titer of sera from rhesus monkeys vaccinated with bivalent STFKB.

(D) The neutralization antibody titer of sera from rhesus monkeys vaccinated with bivalent STFKB after three doses against more recently circulating variants.

(E) The anti-Spike IgG titer of sera from rhesus monkeys vaccinated with bivalent STFKB after three doses

against more recently circulating variants.

The dashed line represents the lower limit of detection. The lower limit of detection was 100 for binding antibody assays and 30 for neutralizing antibody assays. The time point shown (D) indicates the weeks after primary immunization. For intergroup statistical comparisons (B and C), Dunnett's multiple comparison test was used. Asterisks demonstrated statistical significance (**** $p < 0.0001$, *** $p < 0.001$, ** $p < 0.01$, ns indicated not significant).
